# Enhancing micronutrient bioavailability in wheat grain through organic fertilizer substitution

**DOI:** 10.3389/fnut.2025.1559537

**Published:** 2025-04-17

**Authors:** Yafei Wang, Ronghui Ma, Jianlin Wei, Xiaoyan Fu, Shanshan Zhang, Zichao Zhao, Haitao Lin, Yu Xu, Deshui Tan, Xibao Gao, Yumin Liu

**Affiliations:** ^1^Department of Physical and Chemical Inspection, School of Public Health, Cheeloo College of Medicine, Shandong University, Jinan, China; ^2^State Key Laboratory of Nutrient Use and Management, Key Laboratory of Agro-Environment of Huang-Huai-Hai Plain, Ministry of Agriculture and Rural Affairs, Institute of Agricultural Resources and Environment, Shandong Academy of Agricultural Sciences, Jinan, China; ^3^Shandong Agricultural Technology Extension Center, Jinan, China

**Keywords:** organic fertilizer substitution, micronutrients, wheat, bioavailability, nutritional yield, DALYs

## Abstract

The effect of organic fertilizer substitution (OFS) on crop micronutrients often varies due to differences in environmental conditions, soil types, and nutrient status. This study aims to evaluate the effects of OFS on wheat grain micronutrients and bioavailability across five sites in Shandong Province from 2021 to 2022. All experimental sites included five common treatments: control, traditional farming, optimized practices, and 15 and 30% OFS for chemical nitrogen. The results revealed regional variation in wheat yield; the average wheat yield was 9.06 Mg ha^−1^, and the highest yield was 9.58 Mg ha^−1^ in the 15%OF treatment. No significant differences in soil micronutrient availability were observed. Compared to the control, the OFS treatments exhibited significant increases in grain Fe (24.69%) and Zn (19.19%) contents. The OFS treatments significantly increased Fe and Zn bioavailability by reducing the PA/Fe and PA/Zn molar ratios. Organic fertilizer substitution also increased micronutrient nutritional yields and reduced the current health burden of Fe and Zn. Under the pessimistic scenario, the OFS treatment reduced health burdens of Zn and Fe deficiencies by 2.38 and 1.31%, respectively, whereas these mitigation efficiencies substantially increased to 7.15 and 3.94% under the optimistic scenario. In conclusion, OFS improved the content and bioavailability of Fe and Zn without affecting yield, which enhanced the nutritional quality of these nutrients, and alleviate the health burden of Fe and Zn deficiency. The findings demonstrate that a 15% organic fertilizer substitution (OFS) optimally enhances wheat grain Fe and Zn bioavailability and nutritional quality while maintaining crop yield, offering region-specific evidence for sustainable agricultural practices to mitigate micronutrient deficiencies and improve human health outcomes.

## Introduction

Micronutrients are vital for the growth and development of plants, animals, and humans (1). Micronutrient deficiencies (MD) may lead to health problems such as underdevelopment, intellectual disability, decreased or even loss of labor capacity, and decreased immunity (2). Inadequate dietary micronutrient intake is a major cause of MD in humans (3). Wheat, one of the world’s three major food crops, plays an important role in ensuring human nutritional health and food security (4). However, the low content of micronutrients in wheat grains has become a limiting factor for human micronutrient supplementation in China (5). The average Fe and Zn content of wheat grain in the North China Plain is 41.7 and 23.7 mg kg^−1^, respectively (5), which are below the WHO’s proposed fortification targets for Fe and Zn in wheat grains (57 and 45 mg kg^−1^, respectively) (6). Therefore, increasing the content of micronutrients in wheat grains to improve human nutritional health is important for ensuring the high-quality green production of wheat and enhancing the health of the human population.

Organic fertilizer substitution (OFS) is an effective measure for improving soil fertility and quality while maintaining crop yields (7, 8). In addition, micronutrients contained in organic fertilizers can affect the micronutrient profiles of crops (9). A long-term experiment conducted in the North China Plain by Li et al. (10) demonstrated that OFS at 70% significantly increased the soil’s available Fe, manganese (Mn), copper (Cu), and Zn content. Zhang et al. (11) also showed that 25 and 50% OFS increased the Zn content in wheat grains by 57–67% compared with chemical fertilizer treatment. However, many studies have shown that organic substitution has no effect on the content of soil or crop micronutrients (12, 13). In fact, the micronutrient nutritional benefits of OFS are often limited by the environment, and the effect varies with soil type, climate, and soil fertility conditions. However, since previous studies have predominantly focused on enhancing crop grain micronutrient content through micronutrient fertilizer application (14, 15), research investigating the effects of OFS on crop micronutrient profiles remains limited and inconclusive. Consequently, multiple trials in diverse settings are needed to clarify the effect of OFS on the micronutrient nutrition of crops.

Phytic acid (myo-inositol 1,2,3,4,5,6-hexakisphosphate, InsP_6_, PA) in wheat grains limits the bioavailability of micronutrients (16). The molar ratios of PA to micronutrients are widely used as a simplified measure of micronutrient bioavailability in the human diet (17, 18). However, the unresolved interaction between OFS-driven micronutrient enrichment and PA-mediated chelation remains inconclusive. Therefore, it is particularly important to study the effect of OFS on micronutrient bioavailability under different soil conditions. Recently, scientists and policymakers have recognized that traditional food production indicators often overlook human nutritional needs, which are crucial for assessing the sustainability of intensive agricultural systems (19). Consequently, new quality parameters such as nutritional yield have been widely used to assess crop productivity based on their nutritional value (20). Disability-adjusted life years (DALYs) are commonly used to quantify the burden of disease and injury in human populations (21). Estimating the health burden of Zn and Fe deficiency using the DALYs equation and evaluating the health impact of OFS grains. This has practical significance for improving the nutritional intake of the population, especially the rural population in general, and for improving overall health.

Here, the effect of OFS on the content and bioavailability of micronutrients was assessed across five experimental sites varying in fertility levels in Shandong Province to ensure the representativeness of the findings. We hypothesized that a suitable ratio of OFS can enhance grain micronutrients bioavailability while maintaining crop yield. The main objectives of this study were to (1) investigate the effect of OFS on the content of micronutrients (Fe, Mn, Cu, and Zn) in soil and wheat grains; (2) assess the effect of OFS on micronutrient bioavailability in wheat grains; and (3) assess the nutritional yield, health impact (the DALYs saved) and economic benefit of micronutrients in wheat grains.

## Materials and methods

### Site description and experimental design

The field trials were conducted in five counties (Cao County, Shen County, Yangxin County, Liangshan County and Yuncheng County) in the major wheat-producing region of Shandong Province during 2021–2022. All experimental sites have a cool-to-warm temperate monsoon climate. The mean temperature and annual precipitation of the wheat growing season in all experiment sites are shown in Supplementary Table S1. The soil types and initial properties of all experimental sites are shown in Supplementary Table S2.

All experiments consisted of five treatments with three replications: (1) no fertilization (CK); (2) farmers’ conventional cultivation pattern (FP), in which the nitrogen, phosphorus, and potassium application rates were determined based on preliminary investigations in each region; (3) modified farmers’ conventional cultivation pattern (OPT), in which fertilization rates were optimized based on wheat nutrient requirements and the local soil nutrient supply capacity; (4) 15%OF, in which organic fertilizer substituted 15% of chemical nitrogen fertilizer; and (5) 30%OF, in which organic fertilizer substituted 30% of chemical nitrogen fertilizer. Individual plots are 4 × 10 m (40 m^2^) in size, with a randomized complete block design. The amounts of inorganic fertilizers used at each experimental site are presented in Supplementary Table S3. The types and nutrient contents of organic fertilizers used at each experimental site are presented in Supplementary Table S4. At each experimental site, 50% N fertilizer (including organic N and urea N) and phosphate and potassium fertilizers were applied basally. Additionally, 50% N fertilizer as urea was applied at the jointing stage of wheat production. The wheat varieties, planting density, and irrigation were in accordance with the recommended local high-yield cultivation techniques. All plots were irrigated before winter and during the wheat stem elongation period. General pesticides were applied in wheat-growing seasons to control disease, weeds, and insects.

### Sampling and analysis

At the harvest stage of wheat in June 2022, soil samples were collected at each site using a stainless-steel auger along an “X” pattern with five borings (20 cm deep) per plot, which were subsequently merged into single samples. Soil samples were air-dried and sieved through an 8-mesh screen for subsequent determination of the pH and the available phosphorus (P), available potassium (K), DTPA-Fe, DTPA-Mn, DTPA-Cu, and DTPA-Zn content. Total nitrogen (N) and organic matter (OM) content of the soil was then measured for soil samples ground and passed through a 100-mesh sieve. A soil pH meter (SevenExcellence, Mettler-Toledo, China) was used to measure soil pH with a soil-to-water ratio of 1:2.5. Available P and available K were determined according to the methods of Murphy and Riley (22) and Walker and Barber (23), respectively. Total N and organic matter in the soil were analyzed using the wet oxidation method with a Vario Max CN instrument (VarioMax CN; Elementar, Langenselbold, Germany). Soil available Fe, Mn, Cu, and Zn (DTPA-extractable fractions) were extracted using diethylenetriamine pentaacetic triethanolamine (DTPA-TEA) buffer (pH 7.3) and quantified by inductively coupled plasma optical emission spectrometry (ICP-OES, ICAP RQ, Thermo, Waltham, MA, United States), with instrument detection limits of 0.003, 0.002, 0.001, and 0.001 mg L^−1^ for DTPA-Fe, DTPA-Mn, DTPA-Cu, and DTPA-Zn, respectively (24). Soil standard samples (ASA-15) were used for quality control.

At wheat maturity, a central 4 m^2^ (2.0 m × 2.0 m) area of wheat and a randomly selected sample of adjacent wheat plants (0.5 m in length) were harvested in each plot to determine the wheat yield and grain micronutrient content of each plot, respectively. Samples were taken from areas with uniform density, growth height, and growth stage to reduce sampling variation. Deionized water was used to wash the grain samples and dried at 60–65°C until a constant weight was achieved; they were then ground into powder for micronutrient analysis using a stainless steel mill. The samples were subjected to microwave-assisted digestion using a HNO₃-H₂O₂ mixture in closed digestion vessels (CEM, Matthews, NC, United States). The contents of Fe, Mn, Cu, and Zn in the resulting digestates were quantified by ICP-OES (ICAP RQ, Thermo, Waltham, MA, United States), with instrument detection limits of 0.003, 0.002, 0.001, and 0.001 mg L^−1^, respectively. Standard samples (IPE684) (Wageningen University, Netherlands) were used to monitor the quality of the results. The phytic acid content was determined calorimetrically (at 519 nm) as described by Reichwald and Hatzack (25).

### Calculation

#### Nutritional yield

The nutritional yield (NY) denotes the number of adults per hectare per year that can be fully satisfied with their recommended dietary reference intake (DRI) of a specific nutrient from a given crop (20). The NY (adults ha^−1^) is calculated as shown in the following equation:


$$NY=C^{*}GY/DRI/365$$

where C is the content of micronutrients in grains, GY is the dry weight of harvested grains per hectare, and DRI is the daily recommended dietary intake. The calories per 100 g of wheat are 339 kcal (26). The DRI of energy, Fe, Mn, Cu, and Zn are 8.47 MJ day^−1^, 13.00, 2.05, 0.90 and 9.50 mg day^−1^, respectively (27, 28).

#### Health and economic impact


$$\begin{array}{l} \mathrm{Health impact}=\mathrm{Health burden saved}\;by\;OFS\\ \;/\mathrm{Health burden without}\;OFS\times 100. \end{array}$$

In this equation, health burden is equivalent to DALYs. The current health burden (the DALYs lost) was calculated based on De Steur et al. (29). Zn and Fe are predominantly stored in the wheat bran, which gets removed during the wheat processing stage. Merely 31.0% of Zn and 21.7% of Fe are recovered into the grain (30). Consequently, the daily Zn and Fe intake obtained from OFS grains is equivalent to the sum of the status quo daily Zn and Fe intake and the additional total daily Zn and Fe intake, with the latter being calculated by taking into account the recovery rate. Details regarding the daily consumption of wheat (31), the status quo of the daily intake of Zn and Fe (32), as well as the recommended nutrient intake (RNI) (6, 33) are presented in the Supplementary Table S5. In this study, two coverage rates were defined: 20% in the pessimistic scenario and 60% in the optimistic scenario. To evaluate the potential health impacts of OFS wheat grains, the health benefits, represented by the DALYs saved, were calculated. This calculation was based on the increased Zn and Fe contents and was carried out through the methodologies proposed by De Steur et al. (29) and Liu et al. (31).

The following equation was utilized to calculate the economic benefit of OFS wheat grains:


$$\begin{array}{l} \mathrm{Economic benefit}=\mathrm{Total DALYs saved}\\ \;\times \mathrm{PCNI}-\mathrm{Organic fertilizer cost}\text{.} \end{array}$$

where PCNI refers to the per capita net income of China, which is based on a previous study (34). Since the labor required for the application of organic and chemical fertilizers is comparable, in the current study, only the cost of organic fertilizer (900 RMB ha^−1^) was taken into account, in accordance with the survey results.

### Statistical analysis

Data were organized and calculated using Excel 2016. Statistical analyses were conducted using SAS 9.4 software. A one-way analysis of variance (ANOVA) was employed to assess intergroup variability, followed by Fisher’s least significant difference (LSD) *post hoc* test for pairwise comparisons of treatment means. Statistical significance was determined at a predefined level of 0.05, with all hypothesis tests adhering to a two-tailed probability framework. Pearson correlation analysis was conducted using SPSS 27.0 software, with significance levels determined through two-tailed testing. Graphs were made using SigmaPlot 14.0. In order to explore the significant effects of yield and different soil physico-chemical properties on the variation of Fe-Zn content in wheat grains, the Random Forest (RF) method was used to rank the importance of the variables. This step is implemented by R 4.3.1 using the “importance” function in the “randomForest” R package (35). To assess the significance of the variables, we calculated the percentage increase in the mean square error (MSE) (36). The significance of the model and R-squared and the significance of the factors are indicated by R 4.3.1 in the R packages “A3” and “rfPermute,” respectively (37, 38).

## Results

### Spatial distribution of the yield and content of micronutrients

Across all experimental sites, the wheat yields ranged from 5.12 to 11.09 Mg ha^−1^, and the average yield was 9.06 Mg ha^−1^ (Table 1). The regional variation among sites was 7.07–9.85 Mg ha^−1^, with the lowest and highest yields occurring in Yangxin County and Cao County, respectively. The content of micronutrients in wheat grains was variable, with ranges of 19.60–61.94 mg kg^−1^ for Fe, 21.75–50.96 mg kg^−1^ for Mn, 2.23–6.39 mg kg^−1^ for Cu, and 18.40–33.66 mg kg^−1^ for Zn. The mean values of Fe, Mn, Cu, and Zn were 37.13, 31.26, 4.42, and 24.61 mg kg^−1^, respectively. The highest grain Fe, Mn, Cu, and Zn content was observed in Yangxin County, with mean values of 52.86, 41.06, 5.73, and 28.51 mg kg^−1^, respectively. The mean values of the grain Fe, Mn, and Cu content in Shen County and the grain Zn content in Cao County were the lowest (26.48, 27.05, 3.06, and 22.21 mg kg^−1^, respectively). The coefficients of variation (CV) were all greater than 10%; the Fe content was the most variable across all plots, and the Zn content was the least variable.

**Table 1:** Descriptive statistics of the grain yield and Fe, Mn, Cu, and Zn content of wheat.

Parameters	Mean	SD	Median	Range	Regional variation	CV (%)
Yield (Mg ha^−1^)	9.06	1.37	9.47	5.12–11.09	7.07–9.85	15.08%
Fe (mg kg^−1^)	37.13	10.24	34.93	19.60–61.94	26.48–52.86	27.56%
Mn (mg kg^−1^)	31.26	6.10	29.48	21.75–50.96	27.05–41.06	17.64%
Cu (mg kg^−1^)	4.42	1.06	4.23	2.23–6.39	3.06–5.73	23.60%
Zn (mg kg^−1^)	24.61	3.73	23.98	18.40–33.66	22.21–28.51	10.16%

### Effects of OFS on wheat yield and nutritional yield

**Figure 1:**
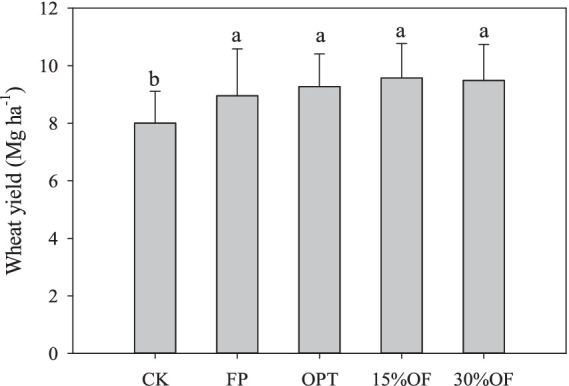
Effect of OFS on wheat grain yield. Columns with the same lowercase letters are not significantly different at *p* < 0.05.

The lowest yield was observed in the CK (8.00 Mg ha^−1^), and the yield was significantly lower in the CK compared with the other treatments (*p* < 0.05, Figure 1). The highest yield was observed in the 15%OF treatment, with an average of 9.58 Mg ha^−1^; the yield was 19.75 and 7.04% higher in the 15%OF treatment than in the CK and FP treatment (8.95 Mg ha^−1^), respectively. There were no significant differences in wheat yield among the other treatments.

Organic fertilizer substitution had similar effects on the nutritional yields of Fe, Zn, and energy in wheat grain. The lowest nutritional yields were observed in the CK, with 53.41, 51.22 and 36.72 adults ha^−1^ for Fe, Zn, and energy, respectively, which was significantly lower than that in other treatments (*p* < 0.05). The highest nutritional yields were observed in the 15%OF treatment, with 78.35, 72.71, and 43.94 adults ha^−1^ for Fe, Zn, and energy, respectively. However, the highest nutritional yield of Mn was observed in the OPT treatment and the lowest nutritional yield of Cu was observed in the FP treatment. This represents significant increases of 19.15, 19.13, and 21.71% for Fe, Cu, and Zn, respectively, in the 15%OF treatment compared with the FP treatment (*p* < 0.05). In all treatments, the nutritional yields of Fe and Zn were 64.87 and 54.09% higher than energy nutritional yield, respectively (Table 2).

**Table 2:** Effects of OFS on Fe, Mn, Cu, Zn, and energy nutritional yields in wheat grains.

Treatments	Nutritional yield (adults ha^−1^)^a^				
	Fe	Mn	Cu	Zn	Energy
CK	53.41 ± 13.04d	328.45 ± 60.50c	110.48 ± 27.60b	51.22 ± 8.33c	36.72 ± 5.06b
FP	65.76 ± 8.23c	374.32 ± 34.07ab	110.45 ± 19.98b	59.74 ± 11.18b	41.08 ± 7.47a
OPT	70.10 ± 12.46bc	401.23 ± 56.25a	118.50 ± 19.87ab	63.80 ± 9.93b	42.55 ± 5.19a
15%OF	80.37 ± 11.53a	397.59 ± 40.98ab	131.58 ± 23.96a	72.71 ± 8.73a	43.94 ± 5.45a
30%OF	77.65 ± 12.95ab	366.18 ± 25.04b	126.54 ± 30.99ab	72.71 ± 11.25a	43.53 ± 5.74a

The values are the means ± SD of five experimental sites.

a The same lowercase letters in the same column indicated that there was no statistically significant difference between different treatments at *p* < 0.05.

### Effects of OFS on soil physicochemical properties and micronutrients in soils and wheat grains

Organic fertilizer substitution had no significant effects on soil OM, pH, total N, available P, available K and DTPA-Fe, Mn, Cu, and Zn contents after wheat harvest (Supplementary Table S6).

**Figure 2:**
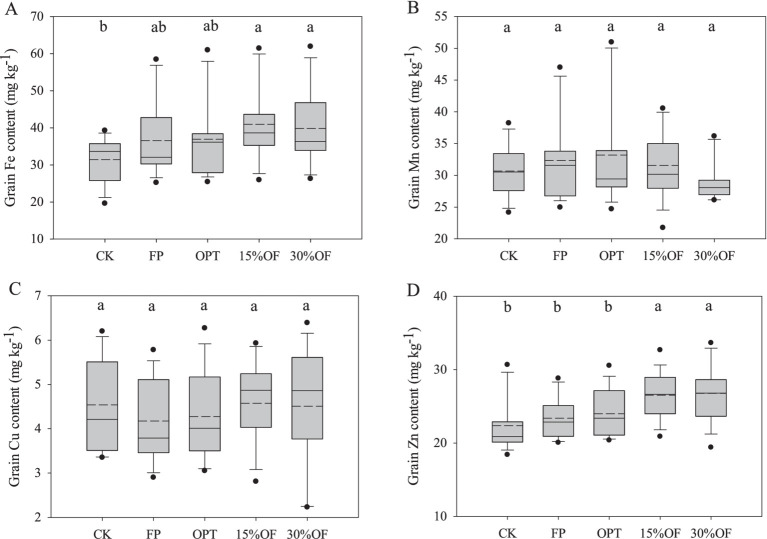
Effect of OFS on the content of micronutrients in wheat grains **(A–D)**. The solid and dashed lines indicate the median and mean values, respectively. Columns with the same lowercase letters are not significantly different at *p* < 0.05.

The Zn content in wheat grain from the organic substitution treatments was significantly higher than that observed in the other treatments (*p* < 0.05, Figure 2). However, no significant difference in the Zn content was observed between the 15%OF and the 30%OF treatments. The wheat grain Fe content of 15%OF and 30%OF treatments were significantly higher than CK treatment (*p* < 0.05), and there was no significant difference between other treatments. The average grain Mn and Cu content among all treatments was 29.24–33.21 and 4.17–4.58 mg kg^−1^, respectively. The organic substitution treatments in this experiment did not significantly affect the Mn, and Cu content in the grain.

### Factors affecting the Fe and Zn content of wheat grain

**Figure 3:**
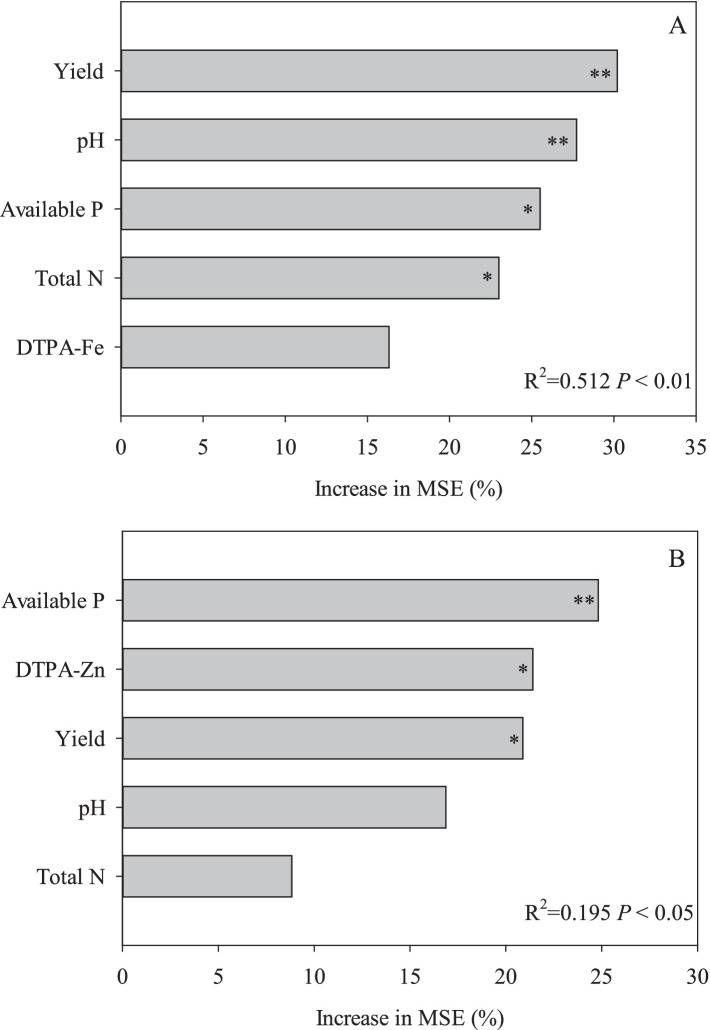
Random forest analysis for grain Fe **(A)** and Zn **(B)** content variations. Increases in MSE (mean squared error) indicate the importance of those variables in the random forest analysis. ^*^p < 0.05 and ^**^p < 0.01.

Random forest analysis showed that yield, pH, soil available P, and soil total N had significant effects on grain Fe content (*p* < 0.05), except for DTPA-Fe (Figure 3A). Whereas, for grain Zn content, soil available P, DTPA-Zn, and yield had a significant effect (*p* < 0.05), pH and soil total N had a non-significant effect (Figure 3B).

### Effects of OFS on the bioavailability of micronutrients in wheat grains

**Figure 4:**
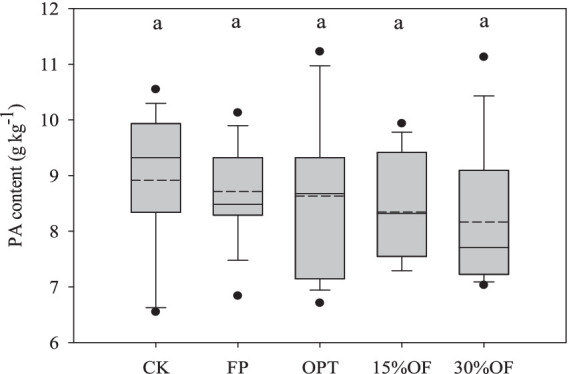
Effect of OFS on the content of phytic acid in wheat grains. The solid and dashed lines indicate the median and mean values, respectively. Columns with the same lowercase letters are not significantly different at *p* < 0.05.

**Figure 5:**
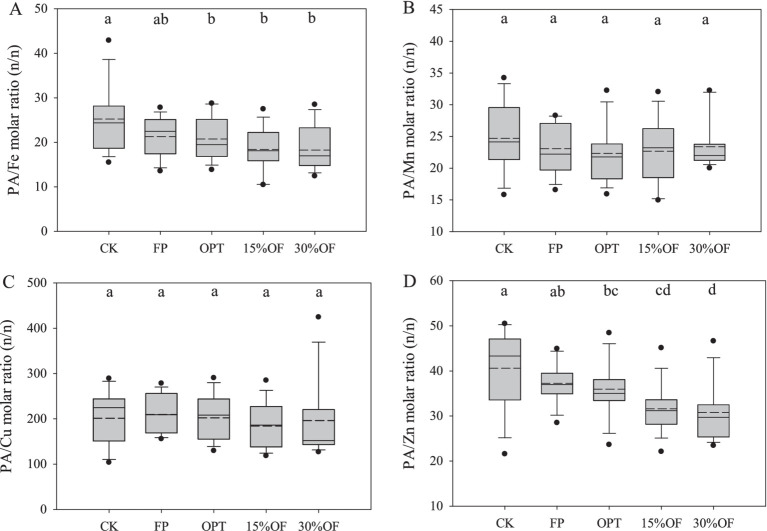
Effects of OFS on the bioavailability of micronutrients in wheat grains **(A–D)**. The solid and dashed lines indicate the median and mean values, respectively. Columns with the same lowercase letters are not significantly different at *p* < 0.05.

The average PA content of each treatment ranged from 8.20 to 8.92 g kg^−1^, and no significant differences in the PA content were observed among all treatments (Figure 4). Organic fertilizer substitution significantly decreased the molar ratios of PA/Fe and PA/Zn in wheat grains in this study (*p* < 0.05). The molar ratios of PA/Fe and PA/Zn were significantly lower in the 15%OF and 30%OF treatments than in the CK, respectively (*p* < 0.05, Figure 5). However, there were no significant differences in the molar ratios of PA/Fe and PA/Zn between the 15%OF and 30%OF treatments. Differences in the molar ratios of PA/Mn and PA/Cu among all treatments were not significant.

### Health impacts of wheat grain with OFS in Shandong Province

Compared to the OPT wheat grain, 15%OF and 30%OF grain increased the daily Zn and Fe intake, the percentage of the RNI, the DALYs saved in the studied population and the potential economic income (Table 3). In the pessimistic scenario, when compared with the OPT grains, the 15%OF and 30%OF grains reduced the current Zn-related health burden in Shandong Province by 2.28 and 2.48% respectively, and the Fe-related health burden by 1.50 and 1.12%, respectively. In the optimistic scenario, the grains in 15%OF and 30%OF treatments also reduced the current Zn-related health burden in Shandong Province by 6.84 and 7.45% respectively, and the Fe-related health burden by 4.50 and 3.37% respectively, compared with the OPT grains. Moreover, in the pessimistic scenario, the economic income generated by the OFS grains could range from 460 to 800 million RMB, while in the optimistic scenario, it could range from 1.4 to 2.4 billion RMB.

**Table 3:** Health impacts of 15%OF and 30%OF wheat grain in Shandong Province.

Parameters	15%OF		30%OF	
	Pessimistic scenario	Optimistic scenario	Pessimistic scenario	Optimistic scenario
Zinc				
Daily Zn intake (mg day^−1^, status quo)				
Infants	4.90	4.90	4.90	4.90
Children	6.00	6.00	6.00	6.00
Daily Zn intake with OFS (mg day^−1^)				
Infants	4.96	4.96	4.97	4.97
Children	6.12	6.12	6.13	6.13
% of recommended nutrition intake with OFS (RNI)				
Infants	71.88	71.88	72.03	72.03
Children	76.50	76.50	76.63	76.63
Health impact (DALYs saved)				
Infants	771	2,313	896	2,689
Children	5,254	15,763	5,673	17,018
% reduction in the current health burden	2.28	6.84	2.48	7.45
Iron				
Daily Fe intake (mg day^−1^, status quo)				
Children <5 years	11.90	11.90	11.90	11.90
Children 6–14 years	18.70	18.70	18.70	18.70
Men 15+	24.40	24.40	24.40	24.40
Women 15+	21.20	21.20	21.20	21.20
Pregnant women	21.20	21.20	21.20	21.20
Daily Fe intake with OFS (mg day^−1^)				
Children <5 years	12.03	12.03	12.00	12.00
Children 6–14 years	18.83	18.83	18.80	18.80
Men 15+	24.66	24.66	24.59	24.59
Women 15+	21.46	21.46	21.39	21.39
Pregnant women	21.46	21.46	21.39	21.39
% of recommended nutrition intake with OFS (RNI)				
Children <5 years	84.13	84.13	83.92	83.92
Children 6–14 years	80.13	80.13	80.00	80.00
Men 15+	90.00	90.00	89.74	89.74
Women 15+	36.50	36.50	36.38	36.38
Pregnant women	36.50	36.50	36.38	36.38
Health impact (DALYs saved)				
Children <5 years	10,211	30,632	7,915	23,744
Children 6–14 years	15,975	47,924	12,337	37,011
Men 15+	49,304	147,912	36,519	109,558
Women 15+	9,781	29,342	7,166	21,497
Pregnant women	44	132	32	96
% reduction in the current health burden	1.50	4.50	1.12	3.37
Economic impact (RMB)	8.0 × 10^8^	2.4 × 10^9^	4.6 × 10^8^	1.4 × 10^9^

## Discussion

### Effects of OFS on wheat yield and nutritional yield

The overall average wheat yield observed in this study aligns with the established yield benchmarks reported in prior agronomic research conducted across the North China Plain (39, 40). And the variation in yield among regions can be attributed to differences in climatic and soil conditions of the experimental sites. Organic fertilizer substitution is an effective fertilization measure for promoting crop growth and increasing yield because of its high richness in nutrients and organic matter content (41). Many previous studies have demonstrated that moderate OFS conditions can maintain or increase crop yields (42, 43). However, a high ratio of OFS can lead to a reduction in wheat yields (40). This can be attributed to the slow release of nutrients from high proportions of organic fertilizers, which results in a lack of available nutrients that are unable to meet crop needs (44). A meta-analysis of studies revealed a significant reduction in wheat yield when OFS was 43% or more (45). In the current study, OFS at 15 and 30% did not reduce wheat yield, which was consistent with the results of Moreira-Ascarrunz et al. (46). This phenomenon may be attributed to the short-term nature of the experiment (first-year implementation), where crop productivity remained strongly influenced by baseline soil fertility conditions. Further multi-season trials are needed to assess long-term effects of OFS on agroecosystem dynamics.

Enhancing land use efficiency in food production is essential for providing sufficient calories and micronutrients for the global population given increasing pressure on land resources (20). Nutritional yield serves as a standardized metric to assess the agronomic efficacy of cultivation methodologies across field-and farm-scale agricultural systems, through the quantification of bioavailable micronutrient output per unit land area (46). In this study, the nutritional yields of Fe, Mn, Cu, and Zn in wheat grains exceeded energy needs, indicating that wheat grain consumption can meet both energy and nutritional needs. The nutritional yields of Fe and Zn were significantly higher in the OFS treatments than in the FP treatment, this suggests that OFS can improve the micronutrient output of current food production systems. The widespread adoption of organic substitution techniques can provide valuable insights for integrating diverse agricultural practices to enhance crop quality and human health in the future.

### Effect of OFS on micronutrient content of grains

The bioavailable soil micronutrient pool constitutes a critical agroecosystem functionality indicator, enabling systematic assessment of wheat grain micronutrient density and thereby serving as a predictor for population-level nutritional security indices within spatially heterogeneous regions (47). In this study, the content of DTPA-Fe, Mn, Cu, and Zn in soil was similar to the background content of these nutrients in Shandong Province (48). According to the classification standard of soil available micronutrients in China, the content of DTPA-Fe, Mn, Cu, and Zn in this study was near values indicative of deficiency (48). Organic fertilizer substitution did not affect the soil DTPA-Fe, Mn, Cu, and Zn content in this study, which contrasts with the results of Li et al. (10) showing that organic fertilizer application for 19 years significantly increased the content of soil DTPA-Fe, Mn, Cu, and Zn. This likely stemmed from the fact that short-term organic application did not provide adequate micronutrients for enhancing soil availability.

The grain contents of Fe, Mn, Cu, and Zn in this study were lower than those observed in the North China wheat region (5). This dilution effect is attributed to the significantly higher grain yields in this study compared with the average wheat yield in North China (49). Organic fertilizer substitution significantly affected the wheat grain Fe and Zn content but had no effect on Mn, and Cu levels, which is consistent with the results of Li et al. (12). In this study, OFS did not affect the soil DTPA-Zn content, suggesting that the increase in grain Zn was not due to enhanced soil Zn availability for crop uptake. Organic fertilization may enhance grain Zn accumulation by releasing endogenous Zn and increasing organic acids, with organic acids improving Zn bioavailability through rhizospheric pH reduction (50, 51). Zinc complexes with low-molecular-weight organic acids comprise more bioavailable forms for crop uptake, which facilitates Zn absorption by plant roots and enhances the Zn content in wheat grains (52). Furthermore, specific growth-promoting bacteria and fungi in soil are critically involved in Zn cycling or possess Zn-solubilizing capacity, which is closely associated with soil Zn availability (53). In this study, the highest Fe and Zn content was 67.63 and 70.47% of the biofortification targets established by HarvestPlus, respectively. This indicates that OFS alone is insufficient for addressing crop micronutrient deficiencies. Nonetheless, these findings provide valuable insights for enhancing crop nutritional quality through agronomic practices.

Pearson correlation analysis revealed strong negative correlations between grain Fe content and both yield and soil available P, whereas no significant correlations were observed with Zn content (Supplementary Table S7). However, random forest analysis identified yield and soil available P as key determinants of grain Fe and Zn content. This discrepancy may arise from nonlinear pathways through which yield and soil available P influence Zn uptake, such as threshold effects or interactions with OM. Previous experimental studies have also documented inverse relationships between grain yield and micronutrient contents (54). Meanwhile, the results of Zhao et al. (55) also showed highly significant correlation between Fe and Zn and P content of wheat grains (*R*^2^ = 0.55). Wheat grain Fe and Zn content decreases with increasing soil available P, which may be attributed to a decrease in mycorrhizal infestation of wheat roots due to increasing soil available P (56). Moreover, DTPA-Fe did not affect grain Fe content and DTPA-Zn significantly affected grain Zn content which is similar to the findings of Nikolic et al. (57). This suggests that increasing exogenous Fe inputs will not increase grain Fe levels in fields with low DTPA-Fe levels (58), whereas combined exogenous Zn supplementation can be used as an important way to increase wheat grain Zn levels in fields with low DTPA-Zn levels (59). In fact, grain micronutrient contents are influenced by a synergistic interplay of edaphic properties, fertilization regimes, and climatic variables. Crucially, soil Zn bioavailability and grain Zn accumulation exhibit significant heterogeneity across soil types (60), with such variations being consistently observed under both irrigated and rainfed management systems (61). In future research, the impacts of these factors on micronutrients bioavailability should be comprehensively considered and evaluated.

### Effects of organic substitution on the bioavailability of grain micronutrients

Micronutrient bioavailability more accurately reflects the extent of micronutrient absorption from grains by the human body compared with the content of micronutrients (31). The results of our study showed that OFS significantly reduced the PA/Fe and PA/Zn molar ratios of wheat grain, which was consistent with the findings of Manzeke et al. (62) showing that organic fertilizer reduced the molar ratio of PA/Zn in maize grains. Tura et al. (63) suggested that the micronutrients are better absorbed by the human body when the molar ratios of PA to Zn and Fe are reduced to less than 15 and 1, respectively. In this study, the lowest molar ratios of PA to Zn and Fe were 31.19, and 18.89, respectively. This indicates that while organic substitution can enhance the bioavailability of Zn and Fe in wheat grains, it remains relatively low and is significantly limited. Therefore, reducing the grain PA content while increasing micronutrient concentrations is essential for further improving micronutrient bioavailability.

### Health impacts of OFS wheat grain in Shandong Province

Given the significance of wheat in the dietary structure of North China and the fact that the bioavailability of Zn and Fe OFS grains is markedly higher than that in OPT grains, substituting conventional wheat grains with OFS grains could significantly mitigate Zn and Fe malnutrition. Our findings indicate that, in terms of the human health impact (the DALYs saved), the 15%OF grains have a greater effect on improving Zn-related health, while the 30%OF grains have a greater impact on Fe-related health. Notably, even under the optimistic scenario with a 60% coverage rate, the health impacts are less than those reported in previous studies (64), but may be more realistic because the amount of Zn and Fe carried over from organic fertilizers is limited. Furthermore, considering the large-scale wheat cultivation area in Shandong Province, which amounts to 4.0 million hectares, implementing the OFS strategy can yield relatively high economic benefits. The economic benefits are more pronounced in rural areas of Shandong Province with relatively lower income levels. Overall, these results suggest that, similar to with Zn and Fe biofortification and genetic biofortification in wheat (65, 66), agronomic biofortification with OFS can reduce the health burden attributable to Zn and Fe deficiency in the studied population. Moreover, it has the potential to boost the economic income of rural households in Shandong Province. These findings have alleviated population-wide micronutrient deficiencies through dietary approaches, particularly benefiting populations in impoverished regions where grain-based diets predominate. This study conducted regional-scale validation of organic fertilization strategies in enhancing grain micronutrient enrichment and bioavailability, providing robust scientific evidence to inform integrated agricultural and public health policies, addressing dietary micronutrient deficiencies in specific regions.

Organic substitution is commonly adopted in intensive crop farming systems in China for its synergistic enhancement of crop yields, sustainability, and soil fertility (8, 39). In this study, no significant difference was observed between chemical and organic fertilizer application treatments, suggesting that OFS even at 30% could maintain high wheat yields. And OFS significantly increased the nutritional yield of Fe, Mn, Cu, Zn, and energy. In addition, OFS treatments increased the soil OM content by 6%, which increased soil fertility. Our findings also indicated that OFS increased the grain Fe and Zn content by 10.59 and 13.99%, respectively, compared with the FP treatment. The grain bioavailability of Fe and Zn also significantly increased by decreasing the PA/Fe and PA/Zn ratio by 18.23 and 17.81%, respectively. More importantly, OFS can alleviate Zn and Fe deficiency in the studied population. Our findings demonstrated that 15%OF is more effective than 30%OF. Overall, organic substitution stabilizes yields, enhances soil quality, and improves the nutritional quality of grains. Under China’s Dual Carbon Strategy (Carbon Peak and Carbon Neutrality), the agricultural practice of substituting chemical fertilizers with organic fertilizers has been increasingly adopted as a prevailing approach. This strategy demonstrates significant potential to synergistically enhance the nutritional quality of grain products and safeguard residents’ health through improved micronutrient bioavailability in the region, thereby aligning with sustainable agricultural transitions and public nutrition security objectives.

## Conclusion

We investigated the effects of OFS on the content and bioavailability of grain micronutrients across diverse sites. Organic fertilizer substitution did not increase wheat yield, but it increased the content and bioavailability of Fe and Zn in grains. Additionally, OFS significantly increased the nutritional yields of Fe, Mn, Cu, Zn, and energy. Organic fertilizer substitution also reduces the current health burden and makes an important contribution to human nutrition and health. An OFS ratio of 15% was optimal for balancing crop yield and nutrient quality. Consequently, the 15%OF protocol can be scaled up for broader implementation. Meanwhile, organic fertilizer varieties should be strategically selected based on regional livestock and poultry breeding patterns to minimize input costs while optimizing local sourcing feasibility for farmers. These findings provide valuable insights into OFS from agricultural, nutritional, and health perspectives, which has implications for improving the safety, sustainability, and economics of future crop production. Moreover, future studies should expand trials across diverse soil types and climatic conditions, coupled with mechanistic investigations into how soil physicochemical properties govern grain micronutrient accumulation. Such efforts will clarify environmental modulation of organic fertilizer substitution effects, optimize fertilizer use efficiency, and ultimately enhance the micronutrient density of grains.

## Data Availability

The original contributions presented in the study are included in the article/supplementary material, further inquiries can be directed to the corresponding authors.
