# Supplementary material for: Enhancing micronutrient bioavailability in wheat grain through organic fertilizer substitution
Source: Front Nutr. 2025 Apr 17;12:1559537. doi: 10.3389/fnut.2025.1559537 (PMC12043458; doi:10.3389/fnut.2025.1559537)
Supplement: Supplementary file 1 [file Data_Sheet_1.pdf]

Table S1. The mean temperature and annual precipitation of the wheat growing season in all experiment sites.

	Temperature (℃)	Precipitation (mm)
Cao County	12.50	31.40
Shen County	11.60	34.98
Yangxin County	10.31	40.88
Liangshan County	12.94	37.42
Yuncheng County	12.50	31.40

Table S2. Physicochemical properties of background soil.

Sites	OM ($g\ kg^{-1}$)	pH	Alkali-hydrolyzed N ($mg\ kg^{-1}$)	Available P ($mg\ kg^{-1}$)	Available K ($mg\ kg^{-1}$)
Cao County	13.6	8.2	62.4	16.6	99.3
Shen County	9.25	8.43	18.23	30.50	216.67
Yangxin County	15.20	8.50	76.1	10.21	152.00
Liangshan County	17.90	8.20	118.40	19.80	221.00
Yuncheng County	14.70	8.10	57.60	35.00	81.00

Table S3. The amounts of inorganic and organic fertilizers used at each experimental site.

Sites	Treatments	Chemical fertilizer application ($kg\ ha^{-1}$)			Organic fertilizer application ($kg\ ha^{-1}$)		
		N	P_2O_5	K_2O	N	P_2O_5	K_2O
Cao County	CK	0	0	0	0	0	0
	FP	246.0	132.0	30.0	0	0	0
	OPT	199.5	108.0	60.0	0	0	0
	15%OF	169.6	108.0	60.0	29.9	15.0	29.9
	30%OF	139.7	108.0	60.0	59.8	30.0	59.8
Shen County	CK	0	0	0	0	0	0
	FP	270.0	135.0	135.0	0	0	0
	OPT	210.0	105.0	105.0	0	0	0
	15%OF	178.5	105.0	105.0	31.5	19.6	39.8
	30%OF	147.0	105.0	105.0	63.0	39.2	79.6
Yangxin County	CK	0	0	0	0	0	0
	FP	255.0	138.0	75.0	0	0	0
	OPT	210.0	90.0	75.0	0	0	0
	15%OF	178.5	90.0	75.0	31.5	30.6	81.2
	30%OF	147.0	90.0	75.0	63.0	61.2	162.4
Liangshan County	CK	0	0	0	0	0	0
	FP	250.5	112.5	112.5	0	0	0
	OPT	210.0	120.0	60.0	0	0	0
	15%OF	178.5	120.0	60.0	31.5	24.2	16.5
	30%OF	147.0	120.0	60.0	63.0	48.4	33.0
Yuncheng County	CK	0	0	0	0	0	0
	FP	250.5	112.5	112.5	0	0	0
	OPT	210.0	120.0	60.0	0	0	0
	15%OF	178.5	120.0	60.0	31.5	24.2	16.5
	30%OF	147.0	120.0	60.0	63.0	48.5	33.0

Table S4. The types and nutrient contents of organic fertilizers used at each experimental site.

Sites	Types	N	P	K	OM	Moisture content
		%				
Cao County	Compost	2.00	1.00	2.00	41.20	35.60
Shen County	Chicken manure	2.25	1.40	2.84	45.00	30.00
Yangxin County	Cow manure	1.04	1.01	2.68	36.30	28.10
Liangshan County	Commercial organic fertilizers	1.30	1.00	0.68	43.90	40.00
Yuncheng County	Commercial organic fertilizers	1.30	1.00	0.68	43.90	40.00

Table S5. Wheat consumption and micronutrient intake.

	Population	Daily wheat consumption (g)	Daily micronutrient intake (mg day^{-1})	
			Status quo (mg day^{-1})	RNI (mg day^{-1})
**Zinc**				
Infants	1100000	75	4.9	6.9
Children 1-5 years	5228322	150	6.0	8.0
**Iron**				
Children <5 years	5334737	150	11.9	14.3
Children 6-14 years	9739472	150	18.7	23.5
Men 15+	40298252	300	24.4	27.4
Women 15+	40420258	300	21.2	58.8
Pregnant women	1122578	300	21.2	58.8

Table S6. Soil physicochemical properties and contents of DTPA-Fe. Mn, Cu and Zn in each treatment after wheat harvest. The values are the means ± SD of five experimental sites and are not significantly different at $p < 0.05$ when followed by the same lowercase letter.

Treatments	OM ($g\ kg^{-1}$)	pH	Total N ($g\ kg^{-1}$)	Available P ($mg\ kg^{-1}$)	Available K ($mg\ kg^{-1}$)	DTPA-Fe ($mg\ kg^{-1}$)	DTPA-Mn ($mg\ kg^{-1}$)	DTPA-Cu ($mg\ kg^{-1}$)	DTPA-Zn ($mg\ kg^{-1}$)
CK	17.18±4.66 a	8.46±0.32 a	1.09±0.35 a	18.84±10.20 a	145.87±66.25 a	8.23±1.70 a	12.99±3.34 a	1.63±1.48 a	1.70±1.11 a
FP	17.72±4.72 a	8.54±0.25 a	1.12±0.38 a	22.29±12.14 a	174.47±44.91 a	7.90±1.40 a	11.47±3.41 a	1.66±1.33 a	1.77±0.95 a
OPT	17.95±4.81 a	8.46±0.32 a	1.11±0.35 a	22.25±11.65 a	145.73±50.65 a	8.10±1.27 a	12.91±4.18 a	1.66±1.34 a	1.85±1.29 a
15%OF	18.85±3.19 a	8.45±0.25 a	1.20±0.36 a	20.99±8.82 a	180.07±76.18 a	7.61±1.09 a	12.93±3.96 a	1.60±1.24 a	1.83±0.99 a
30%OF	18.96±4.47 a	8.36±0.44 a	1.14±0.34 a	20.51±9.50 a	137.03±57.56 a	8.22±1.03 a	13.93±5.23 a	1.72±1.31 a	2.15±1.76 a

Table S7. Pearson correlation analysis of yield and soil physicochemical properties and grain micronutrient contents.

	Fe	Zn
Yield	-0.553**	-0.193
TN	0.381	-0.234
OM	0.229	-0.294
pH	-0.011	0.290
AP	-0.594**	-0.129
AK	-0.390	-0.060
DTPA-Fe	0.471*	0.107
DTPA-Zn	-0.589**	-0.008
